# Vertical Right Axillary Thoracotomy for Repair of Ventricular Septal Defects in Infants and Children: Experience with 50 Consecutive Cases

**DOI:** 10.3390/jcdd13030147

**Published:** 2026-03-23

**Authors:** Yasin Essa, Ali H. Mashadi, Joseph Giamelli, Alexander Mittnacht, Mahmoud I. Salem, Sameh M. Said

**Affiliations:** 1 Division of Pediatric and Adult Congenital Cardiac Surgery, Maria Fareri Children’s Hospital, Department of Surgery, Westchester Medical Center, New York Medical College, Valhalla, NY 10595, USAahmashadi01@gmail.com (A.H.M.); 2 Division of Pediatric Cardiology, Boston Children’s Health Physicians, Maria Fareri Children’s Hospital, New York Medical College, Valhalla, NY 10595, USA; 3 Division of Pediatric Cardiac Anesthesiology, Maria Fareri Children’s Hospital, Department of Anesthesiology, Westchester Medical Center, New York Medical College, Valhalla, NY 10595, USA; 4 Department of Cardiothoracic Surgery, Faculty of Medicine, Port Said University, Port Said 42526, Egypt; drmahmoudsalem@gmail.com; 5 Department of Cardiothoracic Surgery, Faculty of Medicine, Alexandria University, Alexandria 21526, Egypt

**Keywords:** minimally invasive pediatric cardiac surgery, ventricular septal defect, right thoracotomy, right axillary thoracotomy, rapid recovery after cardiac surgery

## Abstract

Objectives: Recently, there has been a growing interest in repairing congenital heart defects in children via right axillary thoracotomy. We sought to review our experience with ventricular septal defect closure through this approach. Patients and Methods: This is a retrospective single-center analysis of 50 children who underwent closure of ventricular septal defects via vertical right axillary thoracotomy between March 2018 and February 2024. We reviewed the patients’ characteristics, perioperative and follow-up data. Results: The study included 26 (52%) girls with a median age of 7 (1–132) months. All patients underwent vertical right axillary thoracotomy with no conversion to sternotomy. Membranous ventricular septal defect was the most common diagnosis and was present in 43 (89%) patients. The median cardiopulmonary bypass and aortic cross clamp times were 96.5 (47–157) and 73 (30–114) min, respectively. In 45 (90%) of the patients, a patch was used. No early or late mortality. All patients were extubated in the operating room, and the median length of hospital stay was 2 (1–321) days. One early reoperation for bleeding, and one patient needed a permanent pacemaker. No late reoperations and all patients/parents were pleased with the incision. Conclusions: The outcomes of the right axillary thoracotomy for repairing ventricular septal defects in children are excellent. The approach is safe and is associated with superior cosmetic results and very short hospital stay. It should be strongly considered as an alternate to sternotomy for closure of ventricular septal defects.

## 1. Introduction

There is no doubt that over the last couple of decades, there have been major advancements in minimally invasive approaches to adult cardiac surgery compared to congenital cardiac surgery [1,2]. Congenital cardiac defects can be repaired reliably and reproducibly through a standard median sternotomy approach with an outstanding result; this approach allows optimal access to all cardiac structures with easy initiation of cardiopulmonary bypass through a central arterial and venous cannulation. However, sternotomy scars are there to stay as a reminder and are regarded as displeasing and potentially stigmatizing, with long-lasting psychological distress, especially in female patients [3], affecting their self-esteem, body image, and overall emotional well-being, often leading to a feeling of being different from peers [4]. Alternative approaches to a median sternotomy have been proposed, including mini-sternotomy [5], thoracotomy (anterolateral [6,7], posterolateral [8]). However, these approaches are not ideal because of long-term sequelae, such as the potential for thoracic deformity, visibility of the scar, and impairment of breast development [9]. The motivation for a less invasive procedure than sternotomy does not come only from the desire to have a more cosmetic incision as a result of a hidden scar, but from the potential for shorter hospital stay, rapid recovery, and quicker return to full unrestricted activity. Recent data suggest that the right axillary thoracotomy approach is a safe and reproducible alternative to standard median sternotomy. This approach, so far, has been associated with excellent outcomes in addition to its cosmetic superiority for a wide variety of heart defects [10,11,12,13]. More and more data are proving the value of this approach to the point where it is becoming a routine procedure for several heart defects in many centers worldwide [12]. We have demonstrated the safety and efficacy of the RAT in treating many heart defects recently [11]. At our center, the vertical right axillary thoracotomy (VRAT) has been our routine approach for many STAT I and II and select STAT III defects for the past few years.

## 2. Materials and Methods

We retrospectively reviewed a total of 50 children who underwent surgical repair of different types of VSDs via VRAT between March 2018 and February 2024. Inclusion criteria were isolated VSD and those associated with simple pathology that can be approached through the right chest, such as simple subaortic membrane resection, double-chambered right ventricle, simple mitral valve repair, and pulmonary valvotomy or removal of a previous pulmonary artery band. Those with more complex left-sided pathology, coarctation/arch hypoplasia, and those who need branch pulmonary artery reconstruction were excluded. Electronic medical records were reviewed to obtain patients’ characteristics, perioperative data, echocardiographic data, and follow-up data. The quality of repair was evaluated with intraoperative transesophageal echocardiogram (TEE) as well as pre-discharge and follow-up transthoracic echocardiograms (TTE).

The Institutional Review Board (IRB) at New York Medical College approved the current study (NYMC IRB # 21396, Date of Approval 18 December 2024), and individual patient/parent’s consent was waived due to the retrospective nature of the current study and the minimal to no risk involved.

## 3. Statistical Analysis

Baseline patients’ characteristics are reported as median ± interquartile range for quantitative variables or as frequencies and percentages for continuous variables. All statistical calculations were performed using SPSS 22.0 software for Windows^®^.

## 4. Surgical Technique

**Figure 1:**
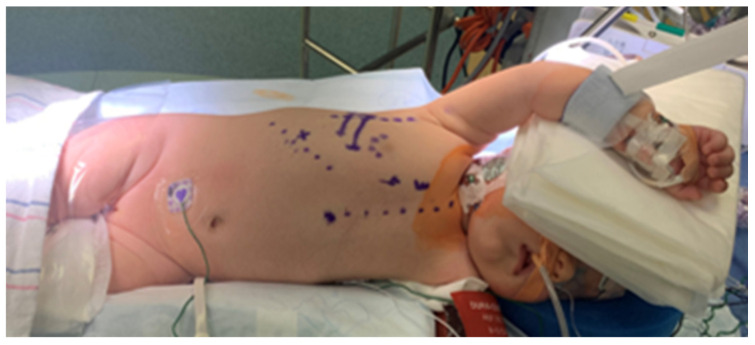
The patient is placed in the modified left lateral decubitus position with the right side up, and the right arm is abducted to expose the axilla. All markings are done while the patient is in the supine position (Solid lines follow the ribs; while interrupted lines point to the sternum, lower rib cage, and the midaxillary line).

We have reported our VRAT technique previously [11]. Briefly, all markings are done while the patient is in the supine position. The patient is then placed in a modified left lateral decubitus with the right side up, and the right arm is protected and abducted above the head to help expose the axillary area (Figure 1). A 4 to 5 cm vertical incision is made in the right mid-axillary line extending from the second to the fifth intercostal spaces. Generous skin and subcutaneous flaps are then created with electrocautery to expose the underlying serratus anterior muscle and the intercostal spaces. Care should be taken to protect the posteriorly located long thoracic nerve (nerve to the serratus anterior muscle).

**Figure 2:**
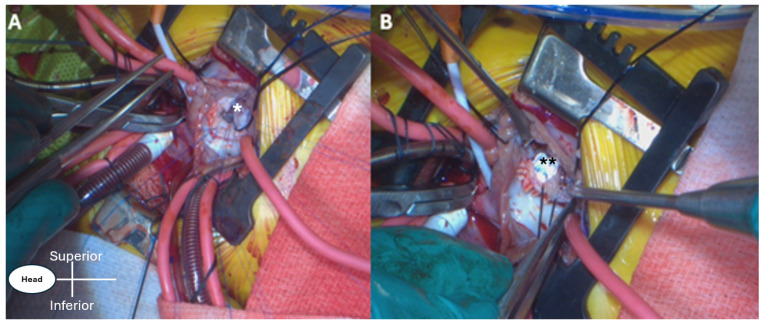
(**A**,**B**) Intraoperative photos showing through the right axillary thoracotomy: (**A**) a large membranous ventricular septal defect (*); and in (**B**) after closure of the defect with the patch secured in place (**).

The incision is a muscle-sparing in nature, which helps with rapid recovery and quick return of arm function postoperatively. For VSD closure, the right chest is entered through the right fourth intercostal space. The lung is then retracted to expose the pericardium, which is incised anterior to the right phrenic nerve, and stay sutures are placed. In the majority of patients, the thymus gland is preserved. In the current patients’ cohort, all cannulation for cardiopulmonary bypass (CPB) is central (aortic and bicaval), and mild hypothermia is usually used. Once cardioplegic arrest occurs, the right atriotomy is created, and the intracardiac anatomy is evaluated (Figure 2A). The VSD is closed in the same fashion as we perform through median sternotomy (Figure 2B), and the tricuspid valve is tested for competency, followed by closure of the right atriotomy, de-airing, and removal of the aortic cross clamp (AXC) with subsequent weaning off CPB, and the rest is done routinely. The pericardial edges are approximated with a few interrupted sutures to prevent adhesions between the right atrial free wall and the medial surface of the right lung. A single chest drain is the routine.

## 5. Results

From March 2018 until February 2024, 50 patients underwent VSD closure. Table 1 shows the clinical and demographic characteristics of all patients. Nearly half of the patients were males 24 (48%). The median age was 7 months (interquartile range, 1–132 months). The median weight was 6 kg (interquartile range, 4–31 kg). There were 12 patients (24%) who had a body weight of <5 kg. Isolated VSD was the most common diagnosis 39 (78%), and membranous VSD was the more frequent category 43 (89%).

Genetic syndromes were encountered in 15 patients (30%), with trisomy 21 being the most common (12; 24%). Two patients (4%) had prior coarctation repair and main pulmonary artery banding via left thoracotomy.

All repairs were done through the axillary approach with no conversion to sternotomy. There was no early/late mortality. Table 2 shows the perioperative data. The median cardiopulmonary bypass time was 96.5 min (interquartile range, 47–157 min), and the median cross-clamp time was 73 min (interquartile range, 30–114 min). Patch was used in 45 (90%) patients, and primary suture closure was done in five (10%).

Concomitant procedures were performed in 12 (24%) patients, including mitral valve repair in four (8%), double-chambered right ventricle repair in four (8%), pulmonary artery de-banding in two (4%), subaortic membrane resection in one (2%), and pulmonary valvotomy in one (2%).

All patients but one were extubated in the operating room and followed an enhanced recovery protocol. There was one preterm patient, who was born at 28 weeks with pulmonary hypertension and spent almost a year in the hospital, and the ventricular septal defect repair was done during his stay.

There was no reoperation for residual defects. Two early reoperations were encountered: one patient developed complete heart block and required placement of a permanent pacemaker (1; 2%), and a second one was taken back due to postoperative bleeding, which was from the chest wall (1; 2%).

The median hospital length of stay was 2 days (interquartile range, 1–321 days). Follow-up was complete with a median follow-up of 17.5 months (interquartile range, 0–72 months). This included routine clinic visits and TTE at the time of hospital discharge, one week, and 3, 6, and 12 months postoperatively, then yearly after.

**Figure 3:**
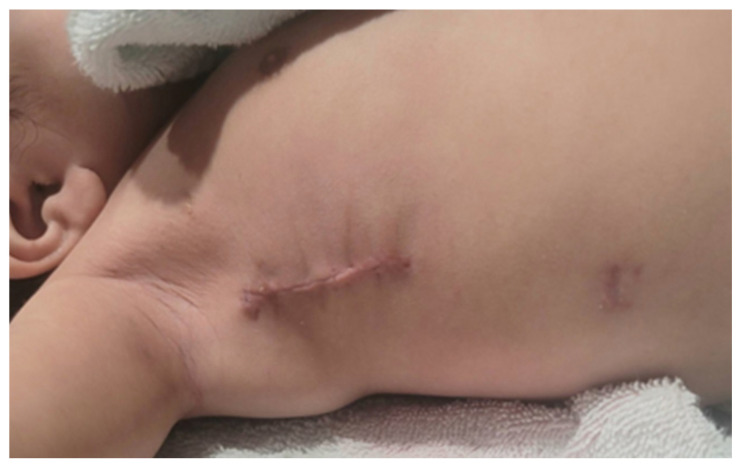
The final appearance of the incision during follow-up where it is completely hidden under the right arm.

One patient developed superior vena cava thrombosis (line-related), which was diagnosed during the first follow-up visit, requiring anticoagulation therapy for 6 weeks as an outpatient, and was entirely resolved on the follow-up. There were no wound-related complications, and all patients/parents were pleased with the scar (Figure 3).

## 6. Discussion

Isolated Ventricular septal defect is the most common congenital cardiac defect [14,15], and thus far, surgery is the gold standard form of therapy. Transcatheter techniques, although they may be suitable for some patients, are not widely adopted in the current era compared to those used in treating atrial septal defects. Potential risks associated with transcatheter device closure of VSDs include device embolization, left ventricular outflow tract obstruction, aortic valve/tricuspid valve leaflet injury, residual shunt, and heart block [16,17].

The main goal behind switching the approach for treatment of these simple congenital cardiac defects from standard sternotomy to axillary thoracotomy is to achieve better cosmesis, rapid recovery with quick return to normal activities [18,19], and minimize the psychological burden of the sternotomy scar and hospital stay on the patients/parents [20]. Rossi et al. have demonstrated that post-traumatic stress disorder was diagnosed in 40% of fathers and 52% of mothers of children who underwent heart transplantation. Their data support the notion that a less invasive perioperative management approach in children with cardiac defects should support both the patients and their parents psychologically [21].

As a result of the growing surgical experience from the atrial septal defect repair, in combination with the gratification of the children, their parents, and our cardiology colleagues, we started using the right axillary thoracotomy approach for the repair of ventricular septal defect at our institution in early 2018. Concerning the setup and instruments used during the right axillary approach, the entire procedure can be done with the same instruments we are using through a median sternotomy.

In our experience, the length of the axillary incision varied between 4 and 5 cm. Indeed, the key to adequate exposure is in part related to the size of the skin flap created and not only the skin incision size. There was no need to enlarge the skin incision, and all cannulations were feasible centrally.

The procedure time, including the cardiopulmonary bypass time and the aortic cross-clamp, is commensurate with that of the standard median sternotomy, which is an indirect measure of exposure adequacy and reproducibility. However, as with any new, unfamiliar approach, a learning curve is required [22]. We believe the majority of isolated VSDs can be closed through this approach, regardless of the size. The subarterial or doubly committed types are challenging, and one should exercise caution when considering VRAT for these types of VSDs. The approach is best suited for membranous, inlet, and inlet muscular types, which constitute the majority of VSDs that require surgical repair. In terms of other selection criteria, regarding the patient’s weight. With more experience, the approach can be offered to small weights (as low as 4 kg), and there is no upper limit to the weight. Regarding associated anomalies, only those that can be addressed through the right side in concomitance with the VSD closure, such as simple subaortic membrane resection, isolated tricuspid or mitral valve repairs, can still be offered to be repaired through VRAT, but arch abnormality and complex left-sided lesions are better approached through standard sternotomy in order not to compromise the repair.

One patient with Down syndrome, status post coarctation repair and main pulmonary artery banding (2%) in our cohort, underwent permanent pacemaker placement. Nonetheless, it is within the average incidence of 1–3% through the standard median sternotomy approach as documented in the literature [23,24].

Right axillary thoracotomy is an example of true rapid recovery after cardiac surgery, but postoperative pain management is critical. Our protocol includes a routine erector spinae block in addition to intrathecal morphine. This results in minimal to no need for postoperative narcotics in our patients. We used intrathecal morphine routinely for every patient preoperatively unless there was a contraindication at the anesthesia team’s discretion [25]. Further interesting observations were the early resumption of oral intake, usually within 4 to 6 h postoperatively, and the faster mobilization, especially in Infants. These observations were reported in the literature by others as well [26]. The median length of stay was two days in the current study. This is the entire duration of stay from the day of surgery. Therefore, we have been discharging the patients home directly from the ICU. A potential explanation has to do with the incision and the postoperative pain. We feel we have comparable cardiopulmonary bypass and cross-clamp times to sternotomy, and the majority of patients are extubated in the operating room in our center regardless of the approach, but VRAT patients are up and more mobile, start feeding or resume oral intake much quicker, and their chest tubes are removed the next day, unlike traditional sternotomy patients. We attribute this to the incision where there is splitting of the sternum vs. no muscle or bone cutting in VRAT.

In terms of the cosmetic aspect of the incision, we received great appreciation and satisfaction from the parents regarding the aesthetic appearance of the incision before the patients’ return to their homes.

In a large series by An and colleagues, 1672 patients underwent repair of CHDs via VRAT. More than half of these patients, 971 (58.1%), had ventricular septal defect repair [12]. The median age and weight were 2.3 (0.2–6) years and 12.5 (5–34) kg, respectively. They had no heart block, and 15 patients were reported to have a trivial residual lesion at discharge with spontaneous closure at follow-up; no mortality was reported. Their skin incision is 1 cm smaller than ours, which can be attributed to the population. Our hospital’s length of stay was a median of 2 days; however, they did not report the length of hospital stay for their patients. One thing we had in common was the satisfaction of patients and their families with incision and cosmetic results.

In the current study, the morbidities and reoperations were quite low. One patient required re-exploration for bleeding, which was found to arise from the chest wall, and a second one needed a pacemaker as mentioned previously. During follow-up, one patient was found to have superior vena caval thrombosis and was treated with anticoagulation for six weeks as an outpatient; and on follow-up, complete resolution of the thrombosis was documented. There was no wound infection or dehiscence in our cohort.

We believe a large body of evidence in the literature demonstrates the feasibility and safety of the axillary approach for repair of a wide variety of CHDs [27]. This supports the need to shift in routine practice from sternotomy to the axillary approach. At the same time, there is no doubt that a learning curve is required, and experience is mandatory as well as visiting centers with expertise in minimally invasive approaches, which is recommended before starting such a program at any institution [28,29].

Study Limitations: We acknowledge the limitations for the current study, which include its retrospective nature, the relatively small number of patients included, and the lack of a control group to compare these outcomes with sternotomy. This, however, will form the basis of future studies related to this topic.

## 7. Conclusions

In conclusion, VRAT provides a safe and feasible approach to repairing VSDs. It provides a combination of excellent outcomes, short hospital stays, and cosmetic superiority that make it an excellent alternative to standard median sternotomy. We anticipate that as experience grows, there will be broader implementation of this approach for patients with more complex defects, such as atrioventricular septal defects, anomalous pulmonary venous connections, and various pathologies of the left ventricular outflow tract.

## Figures and Tables

**Table 1:** Demographic characteristics of the current cohort.

Variable	
Total number	(50)
Sex (Male)	24(48)
Age (months)	7(1–132)
Weight(kg)	6(4–31)
Primary cardiac diagnosis	
Isolated VSD	39(78)
VSD/DCRV	4(8)
VSD/Coarctation	2(4)
VSD/MR	3(6)
VSD/DCRV/SAM	1(2)
VSD/PS	1(2.3)
Type of the VSD	
Perimembranous	43(89)
Inlet	2(4)
Doubly committed	2(4)
Muscular	2(4)
Subarterial(non-committed)	1(2)
Genetic syndrome	
Trisomy 21	12(24)
Trisomy 18	1(2)
Au kline syndrome	1(2)
Ehlers Danlos Syndrome	1(2)
Median Follow up	17.5 months (0–72 months)

Data are median ± interquartile range or *N* (%) of group total, VSD: ventricular septal defect; DCRV: double-chambered right ventricle; MR: mitral regurgitation; SAM: subaortic membrane; PS: pulmonary stenosis.

**Table 2:** Perioperative Outcomes.

Variable	
Total number	(50)
Redo (Yes)	2(4)
CBP time (minutes)	96.5(47–157)
Cross clamp time (Minutes)	73(30–114)
Procedure performed	
VSD patch repair	33(66)
VSD primary repair	5(10)
VSD repair & DCRV repair	4(8)
VSD repair & MR repair	4(8)
VSD repair & PA Debanding	2(4)
VSD repair & DCRV repair& SAMR	1(2)
VSD repair & P valvotomy	1(2)
Repair Type	
Patch	45(90)
Primary closure	5(10)
Complications	
Re-exploration for bleeding	1(2)
PPM placement	1(2)
SVC Thrombus	1(2)
Hospital length of stay (Days)	2(1–321)
Mortality (early & late)	0

Data are median ± interquartile range or *N* (%) of group total, VSD: ventricular septal defect; CPB: cardiopulmonary bypass; DCRV: double-chambered right ventricle; MR: mitral regurgitation; PA: pulmonary artery; SAMR: subaortic membrane resection; P: pulmonary; PPM: permanent pacemaker; SVC: superior vena cava.

## Data Availability

All data in the current study are provided in the current manuscript. Any additional data will be shared on reasonable request to the corresponding author.

## Abbreviations

The following abbreviations are used in this manuscript:

ASD	atrial septal defect
AVSD	atrioventricular septal defect
AXC	aortic cross clamp
CHDs	congenital heart defects
CPB	cardiopulmonary bypass
RAT	right axillary thoracotomy
STAT	Society of Thoracic Surgeons and Congenital Heart Surgeons’ Society Classification
TEE	Transesophageal echocardiogram
TTE	transthoracic echocardiogram
VRAT	vertical right axillary thoracotomy
VSD(s)	ventricular septal defect(s)
